# Near real-time data on the human neutralizing antibody landscape to influenza virus in summer of 2026 shows antigenic advance of H3N2 subclade K region D mutants and H1N1 D.3.1.1 Sa mutants

**DOI:** 10.64898/2026.09.15.751855

**Published:** 2026-11-06

**Authors:** Caroline Kikawa, Andrew Butler, John Huddleston, Sam A Turner, Heidi Peck, Janet A Englund, Kirsten Lacombe, Michael Busch, Marion C Lanteri, Mars Stone, Bryan Spencer, Alexander L Greninger, Derek J Smith, Stephanie Wallace, Helen S Marshall, Shidan Tosif, Scott E Hensley, Ian G Barr, Jesse D Bloom

## Abstract

Human seasonal influenza evolves rapidly, necessitating twice yearly decisions about whether to update the strains in the vaccine. To help inform this decision, we have been using high-throughput sequencing-based neutralization assays to make twice yearly measurements of how recent human sera neutralize current human H3N2 and H1N1 strains. Here we provide the third installment in this series of measurements by reporting 52,268 titers representing neutralization of 148 viral strains by 355 human sera collected between April and August of 2026. Our measurements show that new H3N2 subclade K strains with mutations in antigenic region D and new H1N1 subclade D.3.1.1 strains with mutations in antigenic region Sa (such as G155E) have reduced neutralization by human sera, with notable heterogeneity in the impact of some of these mutations across sera from different individuals. This paper is accompanied by an interactive summary (https://jbloomlab.github.io/flu-seqneut-2026/summary.html) that enables detailed exploration of the results, and all titer data are publicly available for further analysis to aid vaccine antigen selection and studies of viral evolution.

